# A hybrid resistive pulse-optical detection platform for microfluidic experiments

**DOI:** 10.1038/s41598-017-10000-1

**Published:** 2017-08-31

**Authors:** Preston Hinkle, Trisha M. Westerhof, Yinghua Qiu, David J. Mallin, Matthew L. Wallace, Edward L. Nelson, Peter Taborek, Zuzanna S. Siwy

**Affiliations:** 1 Department of Physics and Astronomy, University of California, Irvine, Irvine, CA 92617 USA; 2 Department of Medicine, Division of Hematology/Oncology, University of California, Irvine, Irvine, CA 92617 USA; 3 Department of Molecular Biology & Biochemistry, University of California, Irvine, Irvine, CA 92617 USA; 4 Chao Comprehensive Cancer Center, University of California, Irvine, Irvine, CA 92617 USA; 5 Department of Chemistry, University of California, Irvine, Irvine, CA 92617 USA; 6 Department of Biomedical Engineering, University of California, Irvine, Irvine, CA 92617 USA

## Abstract

Resistive-pulse sensing is a label-free method for characterizing individual particles as they pass through ion-conducting channels or pores. During a resistive pulse experiment, the ionic current through a conducting channel is monitored as particles suspended in the solution translocate through the channel. The amplitude of the current decrease during a translocation, or ‘pulse’, depends not only on the ratio of the particle and channel sizes, but also on the particle position, which is difficult to resolve with the resistive pulse signal alone. We present experiments of simultaneous electrical and optical detection of particles passing through microfluidic channels to resolve the positional dependencies of the resistive pulses. Particles were tracked simultaneously in the two signals to create a mapping of the particle position to resistive pulse amplitude at the same instant in time. The hybrid approach will improve the accuracy of object characterization and will pave the way for observing dynamic changes of the objects such as deformation or change in orientation. This combined approach of optical detection and resistive pulse sensing will join with other attempts at hybridizing high-throughput detection techniques such as imaging flow cytometry.

## Introduction

Resistive pulse (RP) sensing is a method for determining particles’ physical properties by way of measuring the change in resistance of a conducting channel as the particles pass through it^1–5^. Each particle’s passage through the channel results in a measurable decrease in the ionic current, or a ‘pulse’, which can be analyzed to obtain information about the particle’s concentration, volume, charge, and shape^1, 6, 7^. RP can be employed at many scales and in a diverse number of applications, including DNA sequencing (pore diameter ${\mathscr{O}}(1{\rm{nm}})$ ^8–12^, virus detection and characterization (pore diameter ${\mathscr{O}}(10{\rm{nm}})$ ^13–16^, and blood cell counting (channel width ${\mathscr{O}}({10}^{4}{\rm{nm}})$)^1^, and is therefore a very powerful method of analyte sensing. Additional advantages of RP include the ability to measure particles individually, a high-throughput of measurement, a relatively low-cost setup, and low computational complexity. RP is perhaps most useful in systems below 200 nm in size, in which conventional optical microscopy is prohibited by diffraction. Another high throughput single particle characterization system is flow cytometry^17^, where a single stream of cells is analyzed *via* a combination of fluorescent detection and light scattering. In contrast with RP, in flow cytometry unlabeled sub-100 nm objects are difficult to resolve and the measured size is dependent on the refractive index^18^.

To first order, the mechanism for the RP current pulses is straightforward: when a particle enters a conducting channel it occupies a volume that would otherwise be occupied by conductive solution, increasing the channel’s resistance^2, 3^. According to this simple model, the resistance of the channel is determined by the equation $R=\rho \int {A}^{-1}\,(x)dx$ where *ρ* is the resistivity of the solution and *A*(*x*) is the annular cross-section of the channel at axial position *x*. Typically in RP experiments the current is monitored rather than resistance, so the equation for the resistance is inverted *via* Ohm’s law to obtain the current: Δ*R*/*R*_0_ = Δ*I*/*I*_*p*_ where *I* is the measured current, and the subscripts *p* and 0 refer to the quantity measured with and without the particle present in the channel, respectively. While the previous integral equation provides an approximation for calculating the RP amplitude that may be adequate in some cases, it assumes that the current density is evenly distributed across all annuli in the channel, i.e. it does not take into account the electrostatic boundary conditions at the insulating surfaces of the particle and the channel. The effects of these boundary conditions have a large influence on the measured RP amplitude that is position and geometry dependent, and therefore significant efforts have been directed towards finding more accurate expressions for specific cases. For instance, Smythe calculated the expected RP amplitude for spheres passing along the axis of a long cylindrical channel and arrived at the expression^2, 19^

1 $$\frac{{\rm{\Delta }}I}{{I}_{p}}=\frac{{d}^{3}}{L\,{D}^{2}}{[1-0.8{(\frac{d}{D})}^{3}]}^{-1},$$

where *d* is the diameter of the sphere and *L* and *D* are the length and diameter of the channel, respectively. If the channel geometry is known, equation (1) can be used to size particles of unknown size. Furthermore, the cubic dependence on particle diameter allows RP to be effective at discriminating between particles of similar sizes.

While equation (1) serves as a useful starting point for sizing particles in RP experiments, few experimental platforms adhere exactly to the conditions under which it was derived. For example, RP experiments have been conducted in conical^20^ or extremely low-aspect ratio pores^11, 12, 21, 22^. Furthermore, equation (1) was derived for spheres and extended to cover spheroids^6^, but it is often the case that the analyte of interest is poorly approximated by a spheroidal shape^23–25^. Channel and particle geometry aside, the RP amplitude is expected to depend on the particle’s off-axis position inside the channel, an effect not captured by the above equation which was only derived for on-axis translocations^19, 26, 27^.

The typical route taken in cases where equation (1) cannot fully capture the experimental findings is to devise a model that relates the position of the particle, the particle’s geometry, and the geometry of the channel to the expected RP amplitude. Then, based on the model, conclusions are drawn from the RP signal about the positioning and dynamics of the translocation. As a concrete example, Berge *et al*. observed a positive correlation between the translocation times and RP amplitudes of microparticles driven through channels by Poiseuille flow^27^. The authors reasoned that due to the fluid flow profile of Poiseuille flow, particles which had greater translocation times passed closer to the walls of the channel, and due to the observed correlation, off-axis translocation results in a greater RP amplitude compared to on-axis translocations for the same size particles. The authors then derived an equation relating the passage time Δ*t* to the particle’s lateral displacement *y*, and used that equation to come up with an empirical relationship between the off-axis position and the increase to the RP amplitude. As another example, Tsutsui *et al*.^21, 22^, performed experiments in low-aspect ratio pores and found a significant variance in the pulse shape and amplitude. They argued that the variance could be explained by a trajectory-dependent translocation through the channel and devised a model to relate the trajectory to the RP amplitude.

Both of the above cases use only the one-dimensional RP data set to study the complex dynamics of particle translocations through pores. The authors’ inferences may very well be accurate, but could be validated or clarified by simply directly observing the particle translocation while simultaneously recording the RP data. The difficulty in performing these direct measurements is that RP experiments are often performed at the nanoscale, the scale at which RP is most useful but prohibitive towards *in situ* imaging. Unlike in nanoscale systems, simultaneous RP and optical imaging (IM) is possible at the microscale, and therefore microscale RP experiments augmented with optical detection could enable a better understanding of the positional dependencies of the current amplitudes.

**Figure 1:**
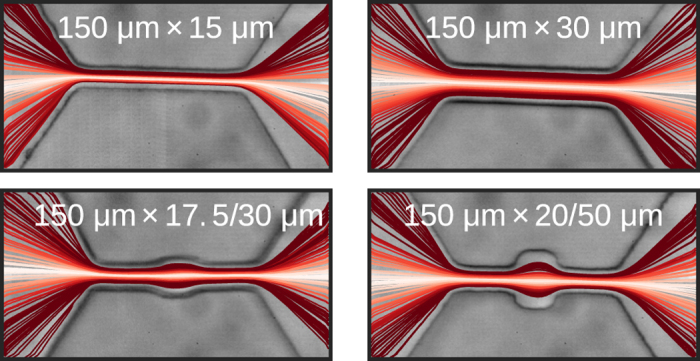
Microscope images of four channels with tracked particle trajectories shown. Each line represents the translocation of a single particle through the channel. Two types of channels, without and with central cavities (top row, bottom row respectively), were studied in this work. Dimensions represent the length and width of the channel. In the case of the channels with cavities, widths of the narrow and wide sections are given. Line color represents lateral displacement from the channel axis. The trajectories indicate that the particles consistently follow streamlines in their approach towards, translocation through, and departure from the channel.

**Figure 2:**
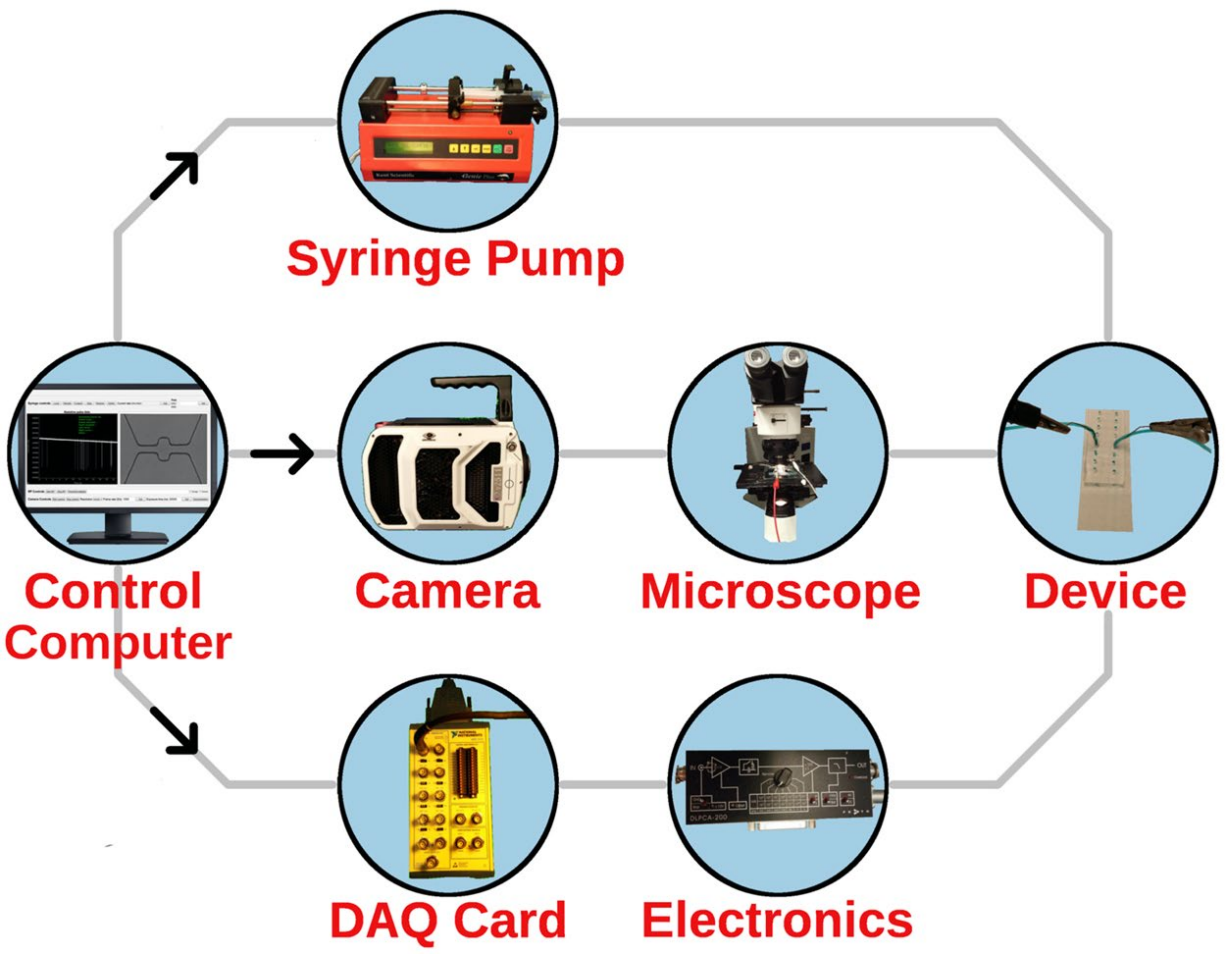
Scheme of the total hardware implementation. A central computer controls the syringe pump, camera, and data acquisition (DAQ) card. Fluid is driven through tubes to the device *via* the syringe pump. A high-speed camera is mounted on to the microscope, and the microfluidic device is placed on the microscope stage. The DAQ card is connected to the electronics in the circuit, which are connected to the device via electrodes.

**Figure 3:**
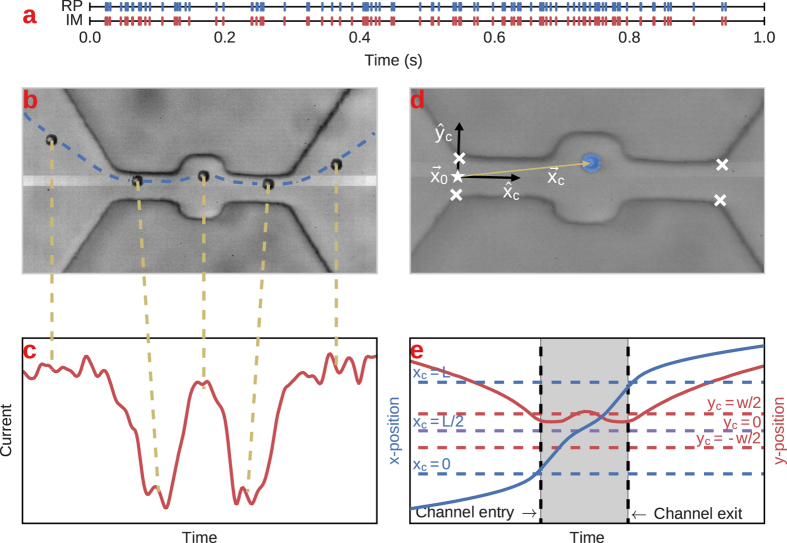
Visualization of a synchronized RP and IM event corresponding to the same particle detection. (**a**) Times of RP (bottom) and image (top) events after alignment. Each line corresponds to a single particle translocation. The events occur over a span of one second. (**b**,**c**) Synchronized RP and image data corresponding to the same particle translocation. The blue dashed line shows the particle’s trajectory through the channel. Gold dashed lines connect the particle’s position and the current amplitude at the same instant in time. (**d**) A coordinate system is defined for each channel based on four corner positions (× symbols). The channel’s origin ${\overrightarrow{x}}_{0}$, denoted by ★, is defined as the average position of the two entrance-side corners, and coordinate axes $\hat{x}$ and $\hat{y}$ are determined by averaging the vectors connecting the corners. The gold-colored vector ${\overrightarrow{x}}_{c}$ is the position of the particle in the channel’s coordinate frame, and is used to create the channel’s resistance map. (**e**) *x*− (blue) and *y*− (red) trajectories of the particle in time. The gray shaded regions indicate the times/positions that the particle was inside the channel. Notice that the particle’s *x*− velocity (slope of *x*(*t*)) is increased inside the channel. The red curve indicates that the particle’s *y*− trajectory followed the contour of the channel edge.

In order to resolve the positional dependence of the RP amplitudes, we devised a microfluidic experimental and data analysis platform that allows simultaneous RP and IM measurements and analysis. The experimental system is based on a high-speed camera (up to 1 MHz) combined with an optical microscope to capture images of particles as they pass through a microfluidic channel while the ionic current is simultaneously recorded (see Figs 1–3). By tracking particles simultaneously with RP and IM and synchronizing the two data streams, we are able to create a position-RP amplitude mapping for each particle. The mappings of many particles are combined to create a ‘resistance map’ of the channel, a two-dimensional plot of the local resistance in the channel as measured by the instantaneous RP amplitude Δ*I*/*I*_*p*_. The resistance map can address specific questions about the positional dependencies of resistive pulses, including:

- At which axial position does the RP amplitude attain its maximal value?
- How does the lateral displacement of a particle inside a channel affect the RP amplitude?
- How is the resistance distributed in variable-width channels?

We note that the idea of combining optical and electronic signals in the resistive-pulse technique was proposed before using fluorescently labeled microbeads^28^. Low spatial and temporal resolution of the imaging signal allowed for recording only one position of the particle for a given translocation, thus an exact mapping of the position with electrical signals was not possible. In this work, we report optical and electrical measurements with 20 *μs* and 4 *μs* time resolutions, respectively. This high time resolution translated into a greater degree of spatial resolution in the position-RP amplitude correspondence measurements; particles traveled short distances relative to the channel’s length between data points. Channels with varying widths were investigated because they emerged as an important analytics tool to extend RP techniques beyond sizing. Indeed, such channels have been already shown to have potential to discriminate objects based on shape and mechanical properties^29–31^. However, resistive pulse experiments with varying width channels are poorly understood, and unlike in particle sizing in cylindrical channels, there is no direct correspondence between the resistive pulse signal and the desired physical properties to be measured. Additionally, the dependence of the resistive pulses on the off-axis position has not been considered for these types of channels. This paper serves as a calibration for these variable width channels, and provides information on the 2D spatial characteristics of RP signals from translocating particles.

We note that augmentation with optical imaging has also been explored for flow cytometry and shown to offer a plethora of additional information not available with the pure method itself^32, 33^. In this manuscript we propose merging RP with imaging to create a very unique platform to dynamically explore properties of particles.

## Results and Discussion

We present experiments with channels that are straight as well as channels that contain one cavity in the middle. Analysis of particle trajectories together with the RP data is presented and discussed for different channel geometries.

### Particle detection coordinate transformation

The ability to determine the particles’ positions with respect to the channel in each frame is absolutely crucial in combining IM and RP signals. We introduce a coordinate transformation that converts the particle’s raw pixel position to a channel-based position measured in physical units. The channel’s coordinate frame is constructed by manually identifying four locations on an image that correspond to the four corner points of the channel. We designate the on-axis position at the entrance of the channel to be the channel’s origin, which is determined from the mid-point of the two corner points closest to the entrance. The channel’s coordinate axes ${\hat{x}}_{c}$ (axial) and ${\hat{y}}_{c}$ (lateral) are also calculated using the corner points. Finally, to convert to units of meters we calculate the ratio of the pixel-length to physical-length of the channel, which is known from the measured dimensions of the mold used to create the channel.

### Event matching protocol

In order to compare the electrical (RP) and imaging (IM) data sets, a protocol must be established for matching events from each data source that correspond to the same particle translocation. First, we construct two time series corresponding to the times of events detected in the RP and IM signals. The times from one signal are shifted until both event sequences overlap. In most instances, there are a few unpaired events that were detected in one time series but not in the other, which are removed. Figure 3a shows the time series for the RP and IM events after alignment, where aligned events represent the same physical particle translocation. At this stage the two sequences are coarsely aligned in such a way that RP event *i* corresponds to IM event *i* and etc., but a fine alignment must still be performed in order to compare individual data points within the matched events. In order to perform this exact matching, we find two data points that are known to correspond to the same instant in time (up to the precision of the camera’s sampling period). For instance, we find the frame where a particle sits at the exact middle of the channel and match it with the middle-most data point in the matched RP event. Fine alignment of the two time series for each event is then achieved by adding a simple time offset to one of the signals, equal to the difference in times of the two corresponding data points. In principle, if both the camera images and current time series recorded at exactly the rates specified by the hardware and did not vary over the duration of the recording, the time offset determined for a single pair of events would perfectly align both the RP and IM time series for their full duration. However, in practice we find a small accumulated discrepancy between the data points over the course of the full recording if only a single offset is applied to match the two data sets. This accumulated discrepancy is insignificant on the time scale of an individual event, but causes a significant mismatch over the time scale of the entire recording. For this reason, a unique offset is calculated for every pair of events compared, and that offset is only used for aligning the time series for that individual particle translocation. Figure 3b,c show the aligned RP and IM signals, where the golden dashed lines connecting the two plots denote data points that correspond to the same instant in time.

### Constant width channels

**Figure 4:**
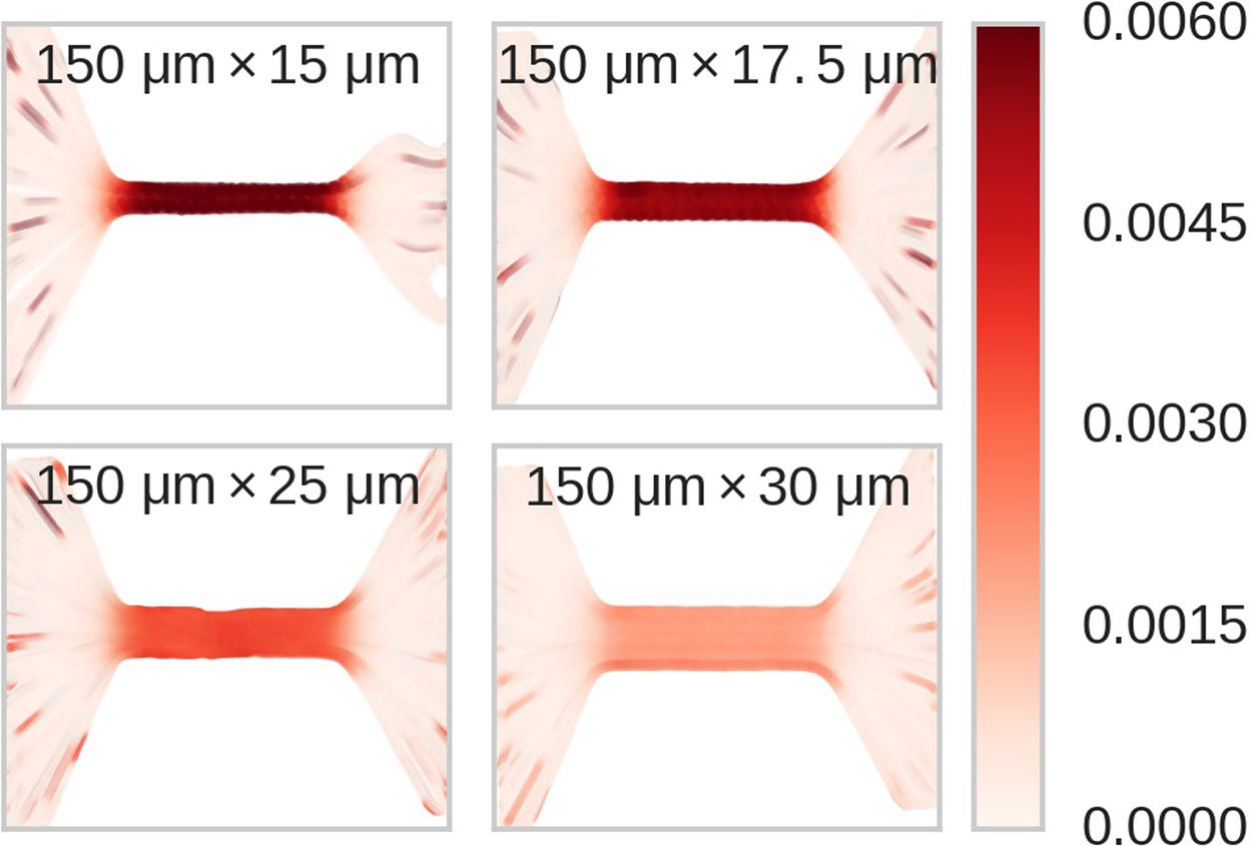
Resistance maps of the four constant-width channels studied in this paper. The colors provide a quantitative description of each system’s resistance. The dark red bands in the middle of each frame represent the resistance of the microchannel, which is far greater than the resistance in the inlet and outlet channels as seen in their lighter red color. Dark red streaks leading up to the channel correspond to brief moments when one particle was approaching or leaving the channel during another particle’s translocation, and therefore do not represent the actual resistance of that part of the channel. The gaps on the right hand side of the 15 *μ*m channel are due to debris outside of the channel, which influenced the particles’ trajectories in this region.

Matching the IM and RP events enables construction of resistance maps, 2D spatial images measured in physical units that are representations of the local channel resistance as measured by the experimental Δ*I*/*I*_*p*_. Each data point in a resistance map represents the instantaneous position of a particle in the channel, and the data point’s color represents the relative RP amplitude Δ*I*/*I*_*p*_ at the same instant in time. Figure 4 shows resistance maps created for straight channels. The interior regions of the channels show relatively darker red colors than the exteriors, indicating a higher resistance. Directly outside the channel, there is a region of moderate resistance known as the access resistance, and a transitional region between the access resistance and the fully developed intrachannel resistance that extends into the channel. In the rest of this section we delve into these aspects of the resistance map in greater detail.

### Position of maximum RP amplitude in the channel

**Figure 5:**
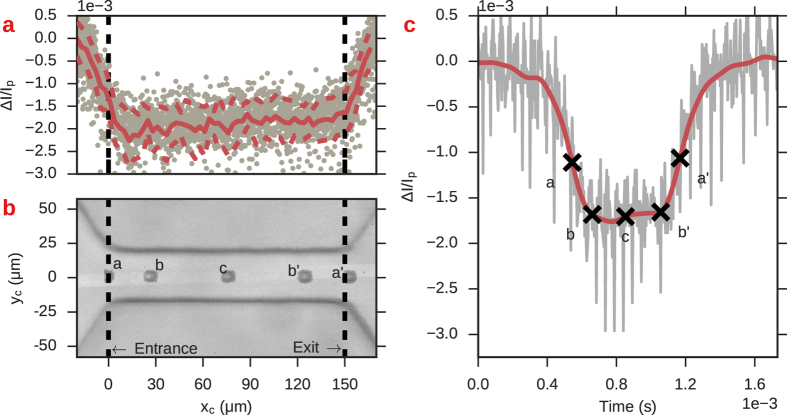
Axial-position *x*_c_ and relative current amplitude Δ*I*/*I*_*p*_ for 10 *μ*m beads passing through a 150 *μ*m in length, 30 *μ*m in width channel. (**a**) *x*_*c*_ and Δ*I*/*I*_*p*_ for multiple events; gray dots represent actual data points. The red solid line is a moving average of data points within ±1 *μ*m of *x*_c_; the width of the window used for the moving average was based on the estimated uncertainty in the measured position of the particle. Red dashed lines are ±1σ from the average Δ*I*/*I*_*p*_. (**b**) Composite image of a single particle translocation. Black dashed lines are the channel entrance and exits. Points a and a’ show the two closest frames corresponding to the particle’s entrance and exit. Points b and b’ are the positions at which the RP amplitude attained its maximal value, as determined by the derivative in the (smoothed) RP signal becoming zero. Point c corresponds to the particle occupying the exact center of the channel. (**c**) Raw (gray) and filtered (red) RP data corresponding to the same particle shown in b. The marked letters correspond to the same instant in time as those marked in b.

In the traditional RP technique, each pulse is analyzed for its duration and amplitude. The pulse duration is a measure of the particle’s velocity, which becomes the basis for measurement of the particle’s ζ− potential^34^. However, precise measurement of translocation time is difficult because there are no standardized locations within a RP signal that are known to correspond exactly to the particle’s entrance and exit. While some studies use the full width at half maximum (FWHM) of the pulse to determine its translocation time^35^, others use the time interval corresponding to the RP current exceeding some value^30, 36^, a threshold that is usually based on some multiplier of the standard deviation of the current. The resistance maps allow us to directly observe the current value corresponding to the entrance and exit points of the particles, and will inform a standard for declaring the event’s beginning and end, and therefore the translocation time, of RP events. We recorded the RP and IM events separately, synchronized them according to the process described above, and used the synchronized data to create a plot of the axial position *x*_*c*_ and instantaneous current value Δ*I*/*I*_*p*_ for each event in the recording. Figure 5 shows a single event from the recorded RP time series alongside the data of many events plotted in coordinates of Δ*I*/*I*_*p*_ − *x*_*c*_. The plots show that there is a significant distance into the channel, greater than the diameter of the particle, before the current amplitude attains its maximal values. This plot suggests that there are transition zones at the entrance and exits of the channel that have lower resistance than at the center, even when the particle is fully encompassed. In Fig. 5b,c, the entrance and exit positions, marked with a, a’, are very close to the FWHM which suggests that using the FWHM to calculate the event duration is more accurate than a threshold approach.

### Dependence of resistive-pulse amplitude on lateral displacement

**Figure 6:**
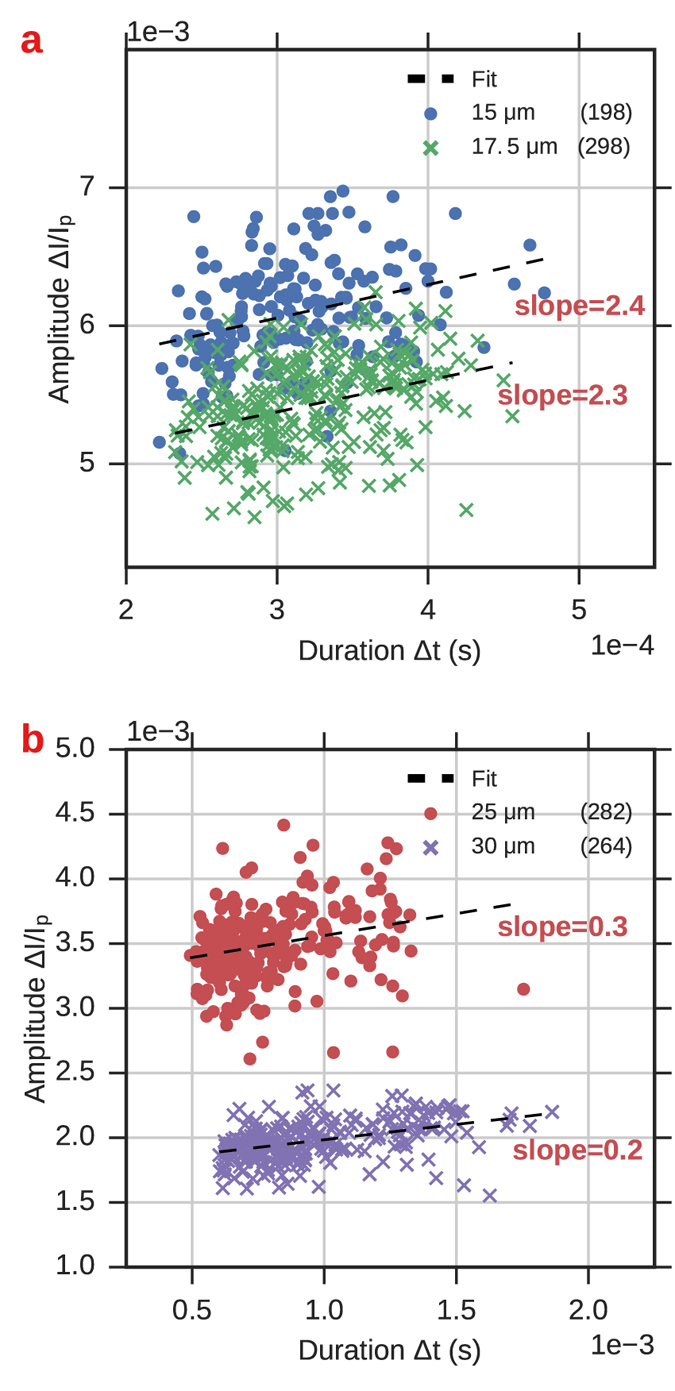
Scatter plot of RP event amplitude versus duration for the four straight channels considered in this paper. Event duration was calculated from the imaging data by subtracting the particle’s channel exit time from its entrance time. Linear fits to the data (dashed lines) show positive correlation between event duration and amplitude. The legend indicates the channel type and number of recorded events for each cluster.

For spherical particles of diameter *d* in cylindrical channels of diameter *D*, the RP amplitude of a particle is given by eq. (1), and therefore measurement of RP amplitudes is an effective means of determining particle diameters^1–3^. However, the equation was derived for particles traveling on-axis, and particles that travel off-axis should have a larger pulse amplitude due to an increased distortion of the electric field in the vicinity of the particle^19, 26, 27^. In a purely diffusive system (e.g. ignoring lateral drift forces), particle trajectories should be approximately uniformly distributed with respect to the channel face, and therefore it is expected that the majority of particles should actually pass closer to the channel surface than on axis. Therefore, understanding the magnitude of the off-axis contribution to the RP amplitude is highly desirable for sizing applications. Berge *et al*.^27^ investigated the off-axis contribution to the RP amplitude by conducting experiments with particles driven through micropores *via* an applied pressure. The distribution of translocation times was found to closely agree with those predicted by Poiseuille flow, and the authors used this fact to infer the particles’ off-axis positions *y*_*c*_ from their passage times. The authors then empirically constructed a relationship between the off-axis position *y*_*c*_ and the recorded RP amplitudes^27^,

2 $$\frac{{\rm{\Delta }}V({y}_{c})}{{\rm{\Delta }}V({y}_{c}=0)}=1+\alpha {(\frac{{y}_{c}d}{D})}^{3},$$

where Δ*V* = *V* − *V*_0_ is the change in voltage (analogous to Δ*I* in our experiments), *y*_*c*_ = 0 indicates a quantity for on-axis translocation, *d* and *D* are the diameters of the particle and channel, respectively, and *α* was a model parameter fit to the data that was found to range from 5–7.5 for the pores used in their study^27^. Saleh *et al*.^26^ extended the work done by Berge *et al*. by noting a linear trend between the pulse amplitude Δ*R* and the translocation time Δ*t*, and that a simple linear correction could be applied to particles based on their translocation time to yield more accurate determination of their size from the RP signal. Figure 6 shows scatter plots of the event duration Δ*t* and amplitude Δ*I*/*I*_*p*_ for the four constant-width channels used in this study. As in the previous studies, we find a positive correlation between event amplitude and duration as shown by the positive slope of the fit to each channel’s scatter data.

**Figure 7:**
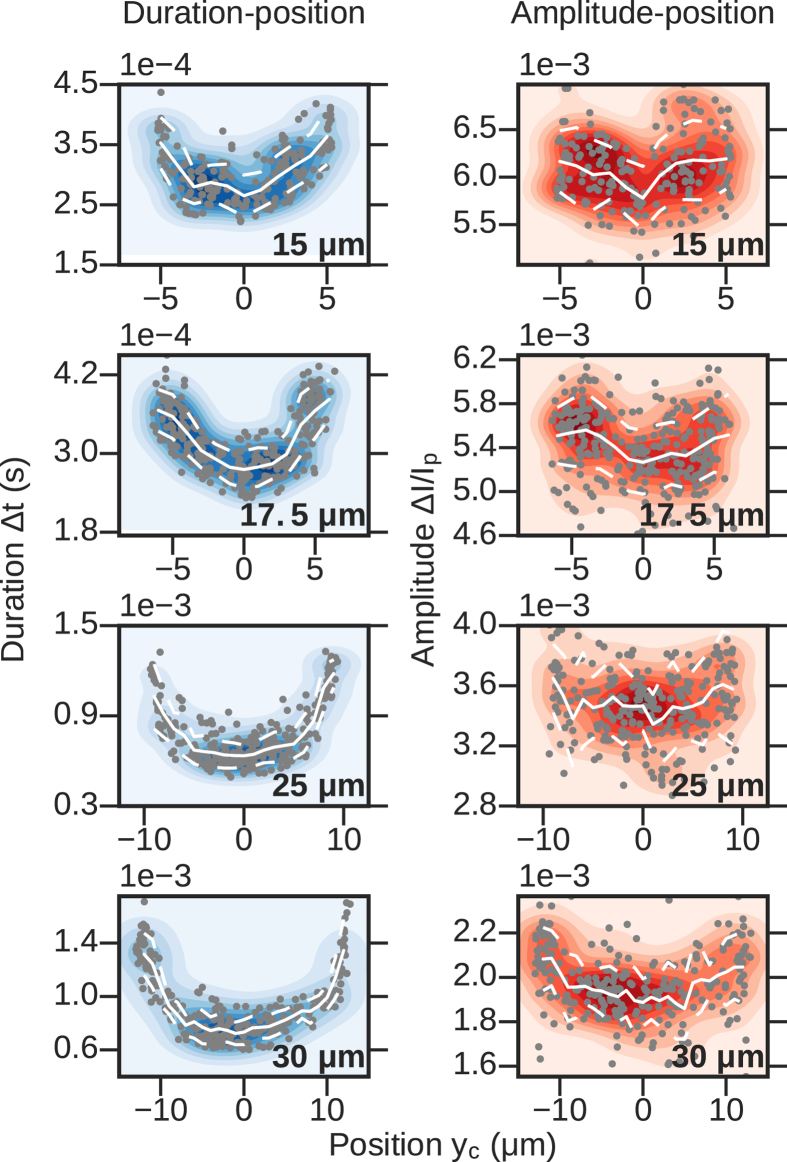
Scatter plots with averages and standard deviations (white lines) and point density (color background). Left: Translocation duration Δ*t* versus off-axis position *y*_*c*_. The fluid flow velocity profile is expected to be described by Poiseuille flow, with a maximum at the center of the channel and minima at the channel surfaces. The event duration is inversely related to the fluid velocity, which is supported by the data. Right: RP amplitude Δ*I*/*I*_*p*_ versus off-axis position *y*_*c*_. Off-axis events are expected to have larger RP amplitudes than on-axis particles, a trend seen in the data for all four channels.

With the simultaneous RP and IM platform, we can directly observe the relationship between off-axis position and event amplitude. The left column of Fig. 7 shows duration Δ*t* vs off-axis position *y*_*c*_ for a few hundred particles passing through each channel. The event duration was determined by the difference in times between the particle occupying the exit and entrance of the channel (the difference in times between *x*_*c*_ = *L* and *x*_*c*_ = 0, see Fig. 3). The off-axis position *y*_*c*_ was calculated by averaging *y*_*c*_ for several individual frames in order to average over the uncertainty in measuring the particle’s exact position in a single frame. When translocating, particles follow axially-aligned streamlines and thus their off-axis positions *y*_*c*_ change very little while they are inside the channel, as evidenced by the trajectories shown in the top row of Fig. 1. The duration-amplitude plots reveal a flow profile qualitatively similar to Poiseuille flow for all four channels, that is, the fits to the translocation times are convex, attaining their maximal values near the channel walls and having local minima at the *y*_*c*_ = 0, or on-axis, position^26, 27^.

The right column of Fig. 7 shows scatter plots of lateral position *y*_*c*_ and relative event amplitude Δ*I*/*I*_*p*_ for the same four channels. The relative amplitude was determined by averaging over multiple data points in the RP signal to smooth over noise; for example, the current *I*_*p*_ was determined by averaging all of the current data points when the particle was within ±50 *μ*m of the channel’s center. All four plots reveal that there is a general trend towards higher event amplitude for translocations occurring closer to the channel walls, however the trend is less pronounced than the translocation duration trend. Applying eq. (2) to the straight channels, we find that the expected excess ranges from 6% to 12% for its maximal value. Although the Δ*I*/*I*_*p*_ − *y*_*c*_ data does not have a perfectly smooth trend, we estimate an excess of roughly 10% for particles translocating off-axis, in agreement with Berge’s findings^26, 27^. However, rather than emphasize the exact quantitative value found, we would like to reinforce that these measurements show an increase in the measured Δ*I*/*I*_*p*_ with increasing lateral position *y*_*c*_, a result only inferred previously. Although the relationship holds for all the channels, it is least pronounced in the 25 *μ*m channel. This could be due to the rougher texture of this channel compared to the others, as evidenced by the wavering streamlines seen in its resistance map (Fig. 4, bottom left plot).

### Channels with cavities

**Figure 8:**
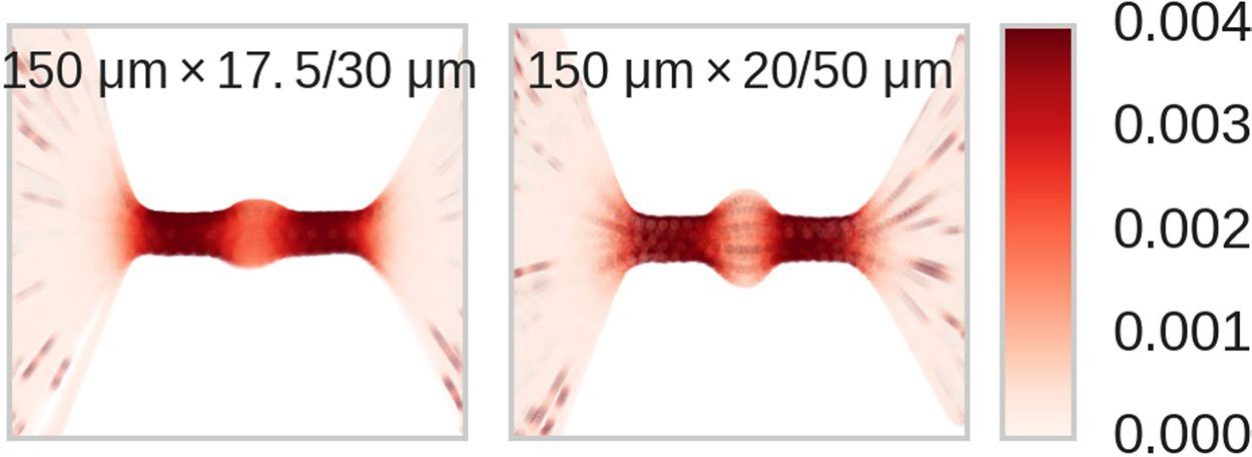
Resistance maps of the two cavitated channels studied shown in Fig. 1. As in Fig. 4, the dark red colors indicate regions of relatively high resistance compared to light red regions. Data points in the channels’ central cavities are lighter than in the narrow regions, indicating smaller Δ*I*/*I*_*p*_ blockages.

**Figure 9:**
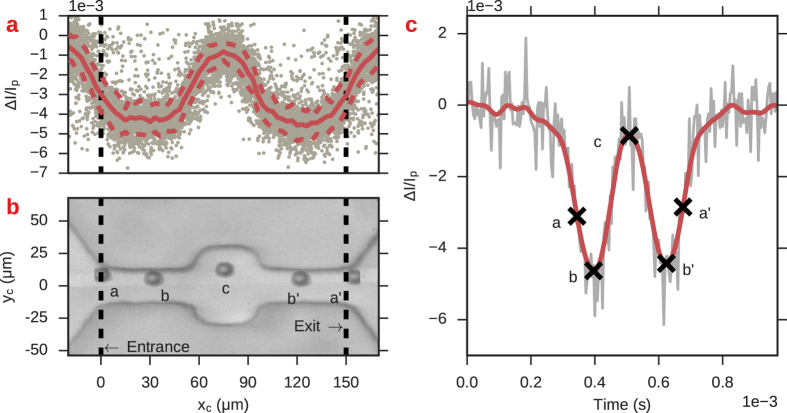
Mapping of axial position *x*_*c*_ to resistance pulse magnitude Δ*I*/*I*_*p*_ in channels containing a central cavity. (**a**) Scatter plot of axial position Δ*x*_*c*_ and relative current amplitude Δ*I*/*I*_*p*_. The solid red line indicates the average of all of the data points at a given position; red dashed lines are the average plus/minus one standard deviation. The black dashed lines indicate the entrance and exit of the channel. The current does not attain its maximal value when the particle occupies the exact entrance/exit of the channel. The local maximum in the center is at positions when the particle occupies the channel’s cavity. (**b**) Superimposed images of the particles occupying various positions in the channel. c: Raw (gray line) and smoothed (red line) RP data of the event passage. The marked positions in b and c mark synchronized data points. a, a’: Closest frames to particle occupying the entrance/exit of the channel. b, b’: Particle position when the current amplitude attains its maximal value. (**c**) Particle position when the current amplitude attains its minimal value.

We next demonstrate the resistance map approach to channels with non-constant cross-sectional areas (Fig. 1, bottom row). Such structures were recently proposed as a platform for probing shape^31^ and mechanical properties of passing objects in a contact-less manner^29, 30, 37^. Figure 8 shows resistance maps of these channels. Similar to the constant width channels, the resistance map reveals a relatively higher resistance in the channel compared to within the bulk, with a transition region in between. The central cavity features a smaller resistance relative to the narrow portion of the channel, as expected from the increased width^36, 38^, and even approaches the baseline resistance value *R*_0_ in the case of the 20/50 *μ*m channel (right side of Fig. 8). Figure 9 shows a plot of Δ*I*/*I*_*p*_ vs axial position *x*_*c*_ along with the current time series of a single RP event for the 20/50 *μ*m channel. The RP event is characterized by two minima and a single maxima, corresponding to the particle occupying the narrow parts of the channel (positions b) and the cavity (position c), respectively.

### Resistive-pulse amplitude in narrow versus wide regions

In cylindrical channels with non-constant radii, the following equation yields the change in resistance of the channel when a particle is present^2^

3 $${\rm{\Delta }}R={R}_{p}-{R}_{0}=\frac{4\,\rho \,{d}^{3}}{\pi D{({x}_{c})}^{4}}{[1-0.8{(\frac{d}{D({x}_{c})})}^{3}]}^{-1}\mathrm{.}$$

Equation (3) is useful because it depends only on the geometry of the channel local to the particle’s position, and is independent of the resistance elsewhere. The formula can also be used to predict the ratio of the relative change in current in one region of the channel to another, since Δ*I*/*I*_*p*_ = Δ*R*/*R*_0_, and therefore

4 $$\frac{{\rm{\Delta }}R{|}_{{x}_{c}={x}_{c1}}}{{\rm{\Delta }}R{|}_{{x}_{c}={x}_{c2}}}=\frac{({\rm{\Delta }}R/{R}_{0}){|}_{{x}_{c}={x}_{c1}}}{({\rm{\Delta }}R/{R}_{0}){|}_{{x}_{c}={x}_{c2}}}=\frac{({\rm{\Delta }}I/{I}_{p}){|}_{{x}_{c}={x}_{c1}}}{({\rm{\Delta }}I/{I}_{p}){|}_{{x}_{c}={x}_{c2}}}\mathrm{.}$$

**Figure 10:**
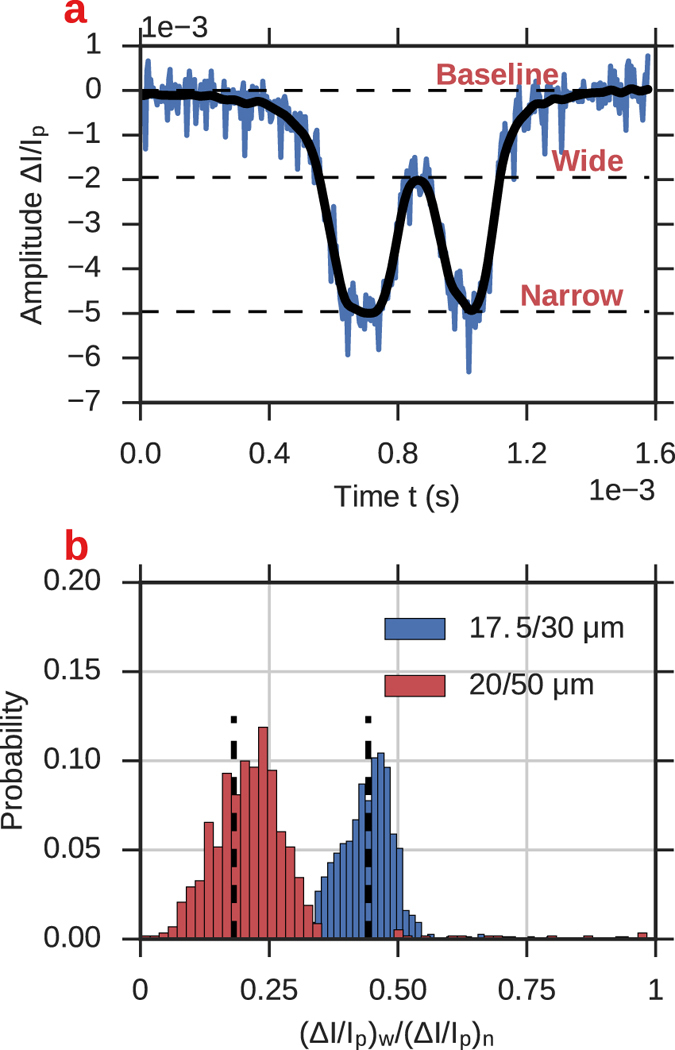
Ratios of the RP amplitude in the cavity and in the narrow entrance of the channel, compared with the theoretical prediction from equation (3). **(a)** A single RP event with dashed lines indicating the relative amplitude Δ*I*/*I*_*p*_ when the particle is outside the channel (‘baseline’), in the central cavity (‘wide’), and in the narrow constrictions (‘narrow’). (**b**) Distributions of ratios of Δ*I*/*I*_*p*_ at the wide and narrow parts of the 17.5/30 *μ*m (red) and 20/50 *μ*m (blue) channels. Dashed lines show the theoretical ratio predicted by equation (3).

Consequently, eq. (4) provides a means to test the accuracy of eq. (3) for our channels by comparing our experimental ratio of relative current amplitudes Δ*I*/*I*_*p*_ with their theoretical value. In order to make the comparison, we approximate our channels as having cylindrical cross-sections with diameters that yield the same equivalent cross-sectional area as our rectangular channels. Figure 10b shows the distribution of ratios of relative current amplitudes in the wide region (the cavity) vs the narrow region (entrance side), along with the theoretical predictions of eq. (3) (dashed lines). For both channels, the theoretical predictions agree well with the experimental distributions and are close to their means, with a relatively larger discrepancy observed in the 20/50 *μ*m channel. This larger discrepancy is likely caused by the cylindrical approximation of the channels, which is expected to be less accurate for the 50 *μ*m cavity, whose width is ~2.5 times greater than its height of approximately 20 *μ*m. We also expect that the resistance of the rectangular geometry will be greater than for the equivalent area cylindrical geometry, which is consistent with the direction of the shift in the distribution of that channel relative to the theoretical prediction. Our results did however confirm the applicability of eq. (3) to describe axial changes of the RP amplitude.

### Dependence of resistive-pulse amplitude on lateral position in narrow and wide (cavity) regions

**Figure 11:**
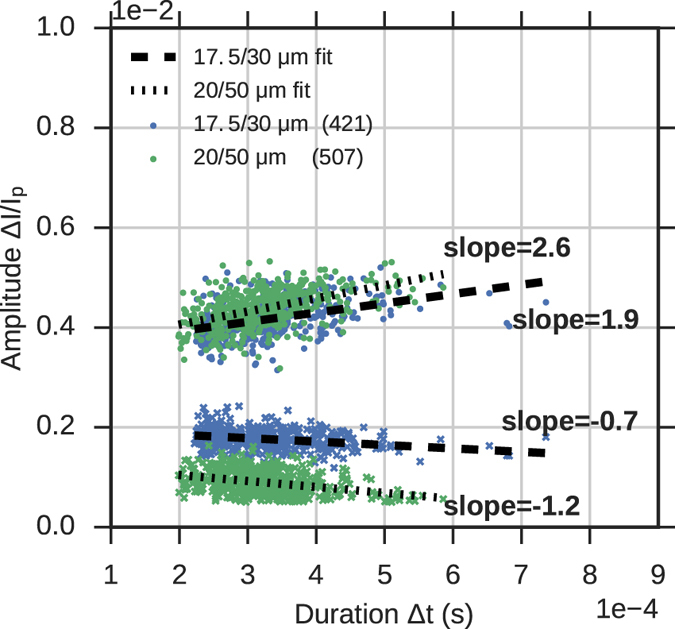
Scatter plot of amplitude and translocation time for particles passing through the channels containing a cavity. For each event we plot the maximum RP current amplitude (when the particle occupies narrow entrance) and the minimum RP current amplitude (when the particle occupies the cavity). The dashed lines show linear fits to the data. The fits show a positive correlation between translocation time and current amplitude when the particle is in the narrow part of the channel, and a negative correlation in the cavity.

**Figure 12:**
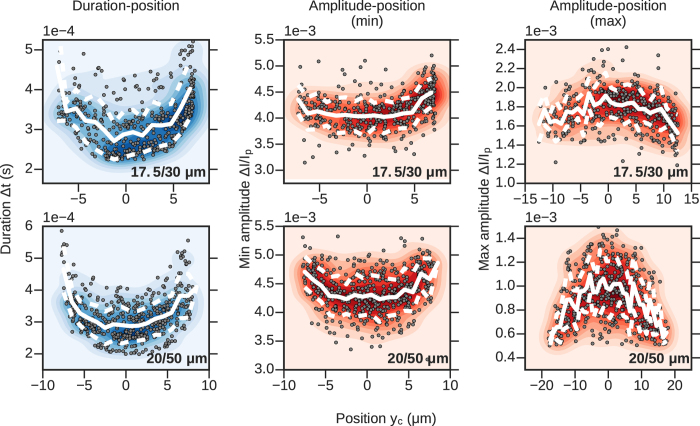
Duration Δ*t* (left column), and relative amplitude Δ*I*/*I*_*p*_ in the narrow (central column) and wide (right column) parts of the channel versus lateral position *y*_*c*_ for two different types of channels with central cavities. Background colors indicate point density; white lines represent point averages and plus/minus one standard deviation from the average. While the general trend in event duration is the same for channels with cavities and straight channels, the dependence of event amplitude on lateral position is inverted between the narrow parts of the channel and the central cavity.

We previously explored the dependence of the RP amplitude on lateral position *y*_*c*_ for straight channels, and now we will reexamine the dependence for when the particle is present in the narrow and in the cavity portions of the channel. As in the previous section, we use the imaging data to measure the lateral position *y*_*c*_ and the current amplitude Δ*I*/*I*_*p*_ from the RP signal at the same time. The results are shown in Figs 11 and 12. In Fig. 11, we again plot amplitude versus duration, but here we include the amplitudes when the particle is in the narrow and in the wide zones, meaning each translocation corresponds to two data points. When the particle occupies the narrow part of the channel, we find the same relationship between duration and amplitude Δ*I*/*I*_*p*_ as we did in the straight channels, namely that the data conforms to a linear fit with positive slope. However, when the particle is inside the cavity we find that duration and amplitude are inversely correlated. This finding suggests that when the particle occupies the cavity, positions closer to the wall have a relatively lower current blockade compared to positions on the axis of the channel. In order to investigate this, we recreate the amplitude-lateral position plots from the straight channel section, shown in Fig. 12.

The leftmost column of Fig. 12 shows scatter plots of translocation time Δ*t* versus lateral position *y*_*c*_ for the two types of channels used in these experiments; here, *y*_*c*_ refers to the lateral position of the particle when it is in the narrow part only. Notice that the relationship behaves as is expected, i.e. is convex upward, meaning that particles passing through the center of the channel translocate much faster than particles traveling along the edges. In the center column, we plot Δ*I*/*I*_*p*_ vs lateral position when the particle is in the narrow part of the channel (position b in Fig. 9). Similar to the previous results, we find that the convex upward shape indicates a larger current blockade for particles traveling at the sides of the channel. Finally, in the third column we show the same plot as in the center column, but for particles in the center of the cavity. Because the cavity has a larger width than the narrow portions of the channel, the *y*_*c*_ data points span a larger range in these plots. Counterintuitively, we find the opposite relationship as in the narrow parts, i.e. that particles passing closer to the axis of the channel have a larger current blockade than particles passing near the sides. The channel proximity effect is in fact much larger in the cavities than it is in the narrow parts of the channel; the difference in the max amplitude between the center and the sides for the 20/50 *μ*m channel is approximately 40%, whereas in the narrow portions we find the excess difference from traveling off-axis is no larger than about 10%, similar to our observations in the straight channels.

We believe the decreased Δ*I*/*I*_*p*_ in positions away from the channel center is due to limited accessibility of this part of the channel to particles. Since particles follow the fluid flow streamlines and they do not travel close to the cavity wall, particles in these locations do not increase the system’s resistance significantly. Our future work will focus on understanding the relationship of the cavity width and the Δ*I*/*I*_*p*_ radial dependence.

## Conclusions

We have presented findings of our experiments on simultaneous RP and optical tracking of individual microspheres passing through microfluidic channels. The experiments provide a means of studying various positional dependencies of the RP experiments that are undetectable with RP alone. The conclusions drawn from these experiments are applicable at the nanoscale, where optical tracking is prohibited due to diffraction. Deviations of the resistance from its ideal form $R=\rho \int {A}^{-1}(x)dx$ are due to the distortion of the electrical field lines imposed by the electrostatic boundary conditions in the system. In the mean-field approximation, the curvature of the warped $\overrightarrow{E}$-fields is scale-invariant, that is, dividing the system size by 10^3^ to translate it from micro- to nanoscale and properly rescaling the voltage will produce the same electric field lines. Therefore, the effects we investigate in this work are also applicable to nanoscale systems. We must note however that at the nanoscale in the presence of surface charges on the pore wall and/or particles, other effects e.g. surface conductance, electroosmosis, and electrophoresis will become prominent^39^. Therefore, we do not suggest that this paper’s results translate directly to nanoscale systems, but rather that the additional nanoscale physics are superimposed with the positional effects, and that ultimately both contribute to the total behavior of the system. Nevertheless, we believe that the results in this work can serve as useful considerations for understanding RP experiments in nanoscale systems, and the general principle of simultaneous RP and IM can be applied to study other important aspects of RP experiments not covered in this work.

Efforts to add a microscopy component to high-throughput measurement systems have so far been focused on imaging flow cytometry (IFC)^32^. In classical cytometry, the data collected consists of detection of scattered light and fluorescence signals; the addition of imaging to the system with IFC provides new information beyond what scattering and fluorescence measurements provide. In contrast to IFC, the dual RP and IM signals described here are complementary to each other, because the electrical RP signals of channels with cavities have already been shown to be sensitive to objects’ physical and mechanical properties. Imaging in our approach is meant to clarify positional effects of the RP signal and to improve its interpretability. This manuscript provides the necessary fundamental information on how position of a particle in pores and channels of various geometries (both axial and radial) affects the resistive pulses. The next step will focus on obtaining similar mappings for aspherical objects and deformable particles. We hope to design an RP device, which for some applications will be able to distinguish objects by shape or mechanical properties based on the electrical signals only. Such particles represent a large share of the particles of interest in biological and medical applications^40–42^.

## Methods

### Microfluidic channel and particle preparation

Microfluidic devices were made of PDMS bonded to a glass slide^43^. The PDMS channels were cast from molds fabricated in a clean room using standard single-step photolithography. SU-8 2025 photoresist (Microchem Corp.) was spun onto a silicon wafer, and patterned using printed transparent photomasks (CAD/Art Services, Inc.). PDMS was then poured over the molds and cured at 75 °C for 3 hours. After curing, the PDMS was removed from the mold, individual devices were cut out, and inlet and outlet access ports for the fluid were punched. Finally, the surfaces of the PDMS devices and glass slides were treated in an oxygen plasma (Harrick Plasma) and subsequently bonded together. Non-deforming polystyrene beads (Bangs Lab) of size 10 *μ*m in diameter and final concentration 2 × 10^8^/mL were used for all experiments. The beads were suspended in unbuffered 1 M KCl. Tween 80, a surfactant that inhibits particle aggregation, was added at 4% concentration to the solution. Particles were micropipetted into the solution and sonicated (Sonic Dismembrator 100, Fisher Scientific) to ensure particles were separated and uniformly distributed. Channel geometries of various lengths and widths were considered, including channels with constant widths and channels which have central cavities (Fig. 1). The channel height was chosen to be ~20 *μ*m to constrain vertical motion as much as possible while preventing particles from getting stuck.

### Experimental hardware

Particle suspensions were driven through the microfluidic devices via a syringe pump (Genie Plus, Kent Scientific), using standard 5 mL plastic luer-lock syringes and polypropylene plastic tubing. A high-speed camera (Phantom v7.2/v2511, Vision Research) was used to capture the particle passages at 50,000 frames per second with 5 *μ*s exposure times, in conjunction with an upright optical microscope (Olympus BX51) and 50x objective lens. Voltages were applied by an Axopatch 200B Patch Clamp Amplifier (Molecular Devices) and the measured current signal was pre-amplified by a low-noise amplifier (DLPCA-200, Femto). Standard Ag-AgCl wire electrodes were used as working and reference electrodes, and were inserted into the system at the inlet/outlet ports. The resulting measured voltage, which is proportional to the current, was sampled by a data acquisition (DAQ) card (National Instruments). A custom GUI program was written in C++ and Qt to enable software control of each instrument and record the current time series and camera images. Communication with each measurement device is unique: the syringe pump is commanded via RS-232 commands over a serial connection, the camera is commanded via TCP sockets over a 1 Gigabit ethernet connection, and the DAQ card is commanded via the National Instruments C API NIDAQmx through a parallel port connection. The instrument control software is open-sourced and available at https://github.com/tphinkle/cell_controller.

### Data analysis and software

A software library was written for analyzing general RP experimental data, and additional code was written to analyze the dual RP/IM data for these experiments. All code, including tutorials written in Python code using Jupyter notebooks, is available open-source at https://github.com/tphinkle/pore_stats. In order to extract the RP events from the measured current baseline, a GUI program was written in Python and PyQt that employs a custom thresholding algorithm. The program includes a number of convenience functions that facilitate easy event detection and validation, including the ability to filter the time series to reduce high-frequency noise and slow baseline drift, and a method for removing groups of events from undesired regions of the amplitude-duration space (e.g. small amplitude and/or duration events arising from noise, or very high-amplitude events caused by particle aggregates). Following event extraction, RP events are processed and analyzed using a library written in Python, which contains functions to remove noise from events, calculate event durations, calculate maxima and minima amplitudes, and generate summary plots. For the imaging analysis, the presence of particles in camera frames was determined by first subtracting the frame from a template known to contain zero particles, followed by pixel intensity thresholding. After thresholding, pixels were grouped into clusters via a flood-fill algorithm. Finally, clusters are tracked across frames using a minimum-distance approach. Clusters of pixels that correspond to the same particle translocation constitute an IM ‘event’, to be matched with a corresponding RP event.

### Data availability

All raw experimental data used in this study is available from the corresponding authors on request.
